# Optimization of Temperature Sensing with Polymer-Embedded Luminescent Ru(II) Complexes

**DOI:** 10.3390/polym10030234

**Published:** 2018-02-26

**Authors:** Nelia Bustamante, Guido Ielasi, Maximino Bedoya, Guillermo Orellana

**Affiliations:** Chemical Optosensors and Applied Photochemistry Group (GSOLFA), Department of Organic Chemistry, Faculty of Chemistry, Universidad Complutense de Madrid, E-28040 Madrid, Spain; nbustamante@aenor.es (N.B.); gielasi@ucm.es (G.I.); mabedoya@ucm.es (M.B.)

**Keywords:** temperature, luminescent sensors, luminescence lifetime, optical fiber, Ru(II) dyes, poly(ethyl cyanoacrylate), water monitoring

## Abstract

Temperature is a key parameter in many fields and luminescence-based temperature sensing is a solution for those applications in which traditional (mechanical, electrical, or IR-based) thermometers struggle. Amongst the indicator dyes for luminescence thermometry, Ru(II) polyazaheteroaromatic complexes are an appealing option to profit from the widespread commercial technologies for oxygen optosensing based on them. Six ruthenium dyes have been studied, engineering their structure for both photostability and highest temperature sensitivity of their luminescence. The most apt Ru(II) complex turned out to be bis(1,10-phenanthroline)(4-chloro-1,10-phenanthroline)ruthenium(II), due to the combination of two strong-field chelating ligands (phen) and a substituent with electron withdrawing effect on a conjugated position of the third ligand (4-Clphen). In order to produce functional sensors, the dye has been best embedded into poly(ethyl cyanoacrylate), due to its low permeability to O_2_, high temperature sensitivity of the indicator dye incorporated into this polymer, ease of fabrication, and excellent optical quality. Thermosensitive elements have been fabricated thereof as optical fiber tips for macroscopic applications (water courses monitoring) and thin spots for microscopic uses (temperature measurements in cell culture-on-a-chip). With such dye/polymer combination, temperature sensing based on luminescence lifetime measurements allows 0.05 °C resolution with linear response in the range of interest (0–40 °C).

## 1. Introduction

Temperature is one of the most important parameters in industrial process control, environmental monitoring, medicine practice, and biology, to name a few areas [1]. Although measurement of temperature by mechanical or electrical methods is widely established, quantification of this physical magnitude can be sometimes extremely difficult due to hostile media for the operator, restricted accessibility, and/or the presence of strong electromagnetic fields that preclude the use of conventional thermometers. Besides, when studying temperature at the micro- and nanoscale, the latter may lack spatial resolution [2]. Commercially available non-contact IR thermometry may be used under some of those particular situations provided that there is a straight free path between the measuring device and the sample point, and the sample is large enough [3]. When these requirements are not met, fiber-optic sensing offers a convenient alternative. Among the various temperature-dependent optical properties that can be used to develop a temperature *optode* (optical sensor), luminescence thermometry, encompassing fluorescence, and phosphorescence, is probably the most sensitive and versatile technique [4], as it shows particular advantages. Firstly, it provides unbeatable spatial resolution, down to intracellular temperature sensing [5,6]; it can also be used for imaging temperature gradients [7,8] and it allows for simultaneous monitoring of temperature and other (chemical) parameters [9,10]. Moreover, luminescence lifetime measurements offer the advantages of being immune to the material photodegradation, indicator leaching, excitation source or detector aging, and to fiber-optic bending.

Many papers and patents have been published about the numerous luminescent materials known, the emission of which is strongly dependent on temperature. Examples are inorganic luminescent phosphors, lanthanide-doped bulk materials, quantum dots, biomolecules, organic molecules, lanthanide chelates, and metal complexes [2,11].

In the last two decades, there has been a strong interest in improving molecular oxygen monitoring using optosensor technologies based on luminescence lifetime quenching of Ru(II) complexes or porphyrins [12]. As a consequence, novel fiber-optic devices have already been marketed for environmental, industrial, or clinical measurements with these robust optodes [13]. Temperature sensing with Ru(II) polypyridyls has also been reported [10,14,15]. It has the advantage of profiting from the vast amount of research and development put forward for fiber-optic O_2_ measurements with such phosphors. Surprisingly enough, it seems that the only temperature indicators of this class used so far have been those already employed for O_2_ sensing [2,7], namely the tris(2,2′-bipyridine)ruthenium(II) [16,17,18,19], tris(1,10-phenanthroline)ruthenium(II) [15,20,21,22,23], and tris(4,7-diphenyl-1,10-phenanthroline)ruthenium(II) complexes [15,24], with the exception of one particular low-temperature application of bis(terpyridine)ruthenium(II) [25]. However, as we will show, the same photochemical features that confer some Ru(II) dyes a good sensitivity to O_2_ [26], make them less sensitive to temperature as well. Moreover, Ru(II) polypyridyls whose luminescence is strongly dependent on temperature, are also the least photostable (see Background section below).

In this paper, we describe how it is possible to design and prepare Ru(II) dyes that are specifically tailored to display a large temperature coefficient for their luminescence intensity and lifetime, while keeping the excellent photostability that characterizes the O_2_ indicator complexes. Moreover, selecting an optimum polymer support for the metal chelate is of paramount importance in order to prevent cross-sensitivity to O_2_ without slowing down the sensor response. The developed temperature optode provides also a way to correct O_2_ (and any other Ru(II)-based) optosensors for the temperature effect by employing the very same instrumentation. The best novel Ru(II) complex has been used here in two different formats, both using a fiberoptic phase shift-based luminometer: (i) as mm-thick polymer monolith onto the optical fiber tips (e.g., for water monitoring applications), and (ii) as μm-thick films into organ-on-a-chip devices, monitored from outside the chip with the optical fibers placed onto the sensitive spots.

**Figure 1:**
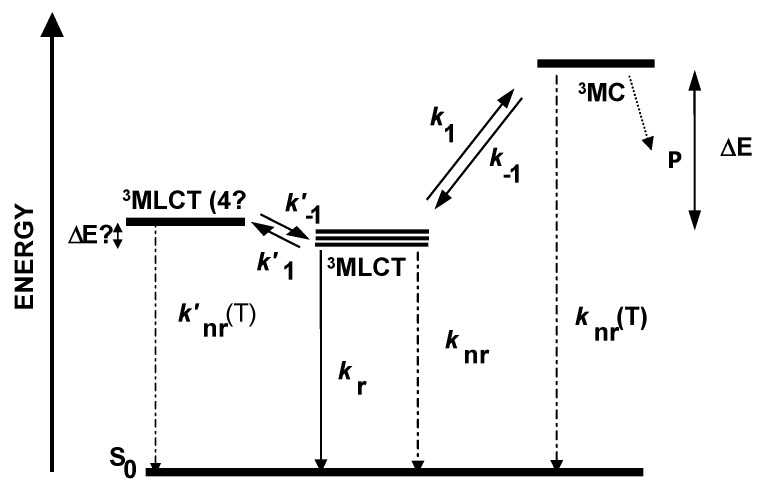
Simplified excited state deactivation diagram for a Ru(II) complex with polyazaheterocyclic chelating ligands. The picture depicts only the emitting and the thermally-activated (dark) excited states.

**Background.** In the absence of emission quenchers, the excited state lifetime of luminescent dyes depends on the competition between radiative and non-radiative deactivation processes. In the case of Ru(II) polypyridyl complexes, it has been demonstrated [27,28,29] that thermal activation can promote the photoexcited electron of the metal dye, located in a luminescent metal-to-ligand charge transfer (^3^MLCT) manifold of three closely spaced states (Figure 1), to a nearby ^3^MLCT excited state (so-called “fourth ^3^MLCT”), and often also to a higher lying non-emissive metal-centered state (^3^MC). The non-radiative constant corresponding to the thermal activation process follows Arrhenius-type kinetics, so that the temperature dependence of the luminescence lifetime (τ, i.e., the inverse of the excited state deactivation rate constant, *k*_d_) for a Ru(II) complex can be expressed according to Equation (1) [30],

(1) $$\tau =1/k_{d}={\left[A+Bexp\left(-\Delta E/k_{B}T\right)\right]}^{-1}$$

where *A* represents the temperature-independent term that includes both radiative and non-radiative deactivation constants from the ^3^MLCT excited state, and *B* is the pre-exponential factor in the Arrhenius equation. The *ΔE* parameter stands for the energy gap (if the crossing is a reversible process) or the activation energy (if it is irreversible) between the ^3^MLCT manifold and the thermally accessible excited state, *k*_B_ is the Boltzman constant, and *T* is the absolute temperature.

From Equation (1), it can be inferred that the temperature sensitivity of the luminescent Ru(II) complexes increases with the accessibility to their ^3^MC state. However, a very efficient crossing to this excited state leads to photolabile species, due to the anti-bonding nature of σ-type ^3^MC states (e(d*)) [31]. The relative energy of the metal-centered level basically depends on the σ-donor features of the chelating ligands (average ligand field strength), while the position of the luminescent ^3^MLCT manifold is determined by the energy of the lowest-lying π* orbital of the ligand set. Therefore, we propose that the synthesis of photostable yet temperature-sensitive Ru(II) indicator dyes should be feasible by selecting appropriate polyazaheterocyclic ligands for engineering heteroleptic (i.e., made of different ligands) metal complexes.

## 2. Materials and Methods

### 2.1. Luminescent Dyes

**Figure 2:**
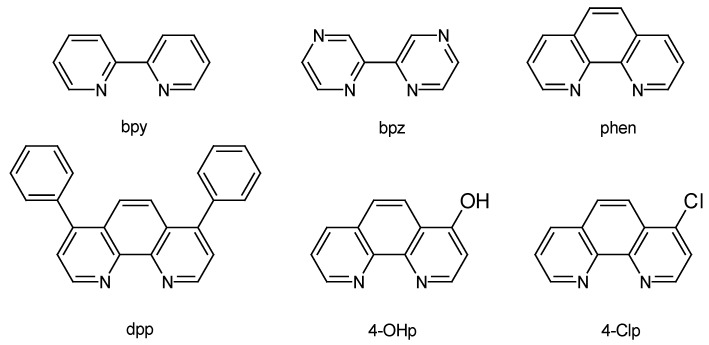
Chemical structure of the chelating ligands used in this work.

The homoleptic complexes tris(1,10-phenantroline)ruthenium(II) dichloride, abbreviated [Ru(phen)_3_]Cl_2_, tris(2,2′-bipyridine)ruthenium(II) dichloride, abbreviated [Ru(bpy)_3_]Cl_2_, and tris(4,7-diphenyl-1,10-phenanthroline)ruthenium(II) dihexafluorophosphate, abbreviated [Ru(dpp)_3_](PF_6_)_2_, were from Sigma-Aldrich (St. Louis, MO, USA) and were used without further purification. Tris(2,2′-bipyrazine)ruthenium(II) dihexafluorophosphate, abbreviated [Ru(bpz)_3_](PF_6_)_2_, was synthesized according to standard procedures [32] by refluxing the corresponding commercial ligand (Sigma-Aldrich, St. Louis, MO, USA) (Figure 2) with RuCl_3_ trihydrate (Alfa Aesar, Karlsruhe, Germany) in ethylene glycol (+99.5%, Panreac, Castellar del Vallès, Spain), and precipitating the PF_6_^−^ salt by adding a concentrated aqueous solution of NH_4_PF_6_ (Fluka, Buchs, Switzerland). Commercial [Ru(bpy)_3_]Cl_2_ and [Ru(phen)_3_]Cl_2_ were metathesized in aqueous solution to their PF_6_^−^ salts by a similar procedure. Preparation of *cis*-Ru(phen)_2_Cl_2_ was carried out, as described in the literature [33]. The heterocyclic chelating ligands 4-hydroxy-1,10-phenanthroline (4-OHp) and 4-chloro-1,10-phenanthroline (4-Clp) (Figure 2) were synthesized following the Snyder et al. procedure [34]. [Ru(phen)_2_(4-OHp)]^2+^ and [Ru(phen)_2_(4-Clp)]^2+^ were obtained by refluxing a 10% excess of the corresponding ligand with *cis*-Ru(phen)_2_Cl_2_ in 6 mM sodium methoxide/methanol (anhydrous) or ethanol/water (5:1 *v*/*v*), respectively [35,36]. The crude complexes were purified by flash chromatography on CM-Sephadex^®^ (GE Healthcare, Chicago, IL, USA), using an aq. NaCl gradient as the eluent. Their chemical structure was confirmed by high-field ^1^H-NMR spectroscopy and MS-ESI spectrometry [35,36].

### 2.2. Solvents and Polymer Supports

Propylene carbonate (PC) was from Merck (+99.7%). Methyl methacrylate (MMA, +99%, Merck, Darmstadt, Germany) was distilled under reduced pressure after removing the polymerization inhibitor it contains by washing with aqueous sodium hydroxide (5% *w*/*v*). Azobis(isobutyro)nitrile (AIBN, +98%, Sigma-Aldrich, St. Louis, MO, USA) was recrystallized from methanol. Ethyl hydroxymethylacrylate (EHMA) was prepared following a reported method [37]. Ethyl 2-cyanoacrylate (“Super-Glue 3^®^”) and two-component epoxi resin (Scotch-Weld DP-100) were from Loctite (Düsseldorf, Germany) and 3M (Maplewood, MN, USA), respectively. Tetraethoxysilane (98%) was from Merck (Darmstadt, Germany).

### 2.3. Spectroscopic Measurements

UV-VIS spectra were collected in a Cary-3Bio spectrophotometer (Varian, Palo Alto, CA, USA). Steady-state emission spectra in solution were recorded at 25 °C in a Perkin-Elmer LS50-B spectrofluorometer. Quartz cells for fluorescence (10 × 10 mm^2^, Hellma, Müllheim, Germany) have been employed for all of the spectroscopic determinations. Time-drive luminescence measurements were performed with a portable fiber-optic spectrometer (Guided Wave model 260), fitted with colored glass band-pass and long-pass filters (Oriel-Newport, Irvine, CA, USA) centered at 400 and 570 nm, respectively. The luminescence quantum yield of [Ru(phen)_2_(4-Clp)]Cl_2_ in deoxygenated water was measured by the Parker and Rees method [38], using [Ru(bpy)_3_]Cl_2_ as reference dye (Φ_em_ = 0.063 [39]).

### 2.4. Luminescence Lifetimes

Emission decay measurements in solution were performed with a single photon timing spectrometer (SPT, Edinburgh Instruments FLS-980, Livingston, UK) equipped with a 450 nm LED head (Nanoled 450L, Horiba, Kyoto, Japan) ns-pulsed at 20 or 40 kHz as the excitation source (through a 467 nm interference filter) and a red-sensitive Hamamatsu R-955 photomultiplier cooled at −20 °C. Solutions of the ruthenium complexes in PC (7–16 μM) were deoxygenated with argon (>99.99%, Praxair, Madrid, Spain), sparging them for at least 30 min before collecting the emission decays. The luminescence lifetimes of the sensor tip were also measured as a function of temperature with the SPT spectrometer. The cuvette sample holder of the FLS-980 was replaced by a homemade fiber-optic adapter to which a (randomly) bifurcated silica waveguide bundle was connected (FiberGuide, Township, NJ, USA, 6.5 and 4.5 mm diameter at the common end and at each branch, respectively). The emission decays were fitted to a single exponential function (solutions) or to the minimum number of summed exponentials (doped polymers) to achieve a reduced χ^2^ value lower than 1.1 using the Edinburgh Instruments proprietary software based on non-linear least squares fitting. Caution must be exercised with multi-exponential decay functions because they might not correspond to the presence of a discrete number of luminescent species, but rather to a plurality of luminophore populations of different widths, as shown by the Edinburgh Instruments (Livingston, UK) advanced Fluorescence Analysis Software Technology (FAST).

### 2.5. Phase-Sensitive Luminescence Measurements

Phase-sensitive monitoring was carried out with a field-deployable fiberoptic phase-sensitive luminometer developed in our research group. The instrument is able to determine simultaneously up to four target parameters (e.g., temperature, O_2_, moisture, CO_2_), or a single parameter in different locations, using common electronic and optoelectronic components, but specific Ru(II) polypyridyls immobilized in tailored polymer supports. The system is equipped with four 470 nm LEDs (9600 mcd, RS Amidata, Madrid, Spain) as excitation light sources, digitally modulated at 39, 78, or 156 KHz (user selectable). The optical module contains a 450 nm CS5-60 colored band-pass filter (100 nm FWHM) (Kopp Glass, Pittsburgh, PA, USA) in the excitation channel, a 503 nm dichroic filter (Edmund Optics, York, UK), and a 570 nm long-pass filter (CS2-73, Kopp), plus a plano-convex lens with 4 mm effective focal length (Edmund) in the emission channel. The excitation light is focused onto a 1000 μm multimode plastic optical fiber (Mitsubishi Cable, Tokyo, Japan) fitted with a SMA connector (Ratioplast Optoelectronics, Lübbecke, Germany). The luminescence from the four sensitive terminals is monitored with an H11901-01 compact photomultiplier (Hamamatsu Photonics, Hamamatsu, Japan). The reference signal is obtained directly from the excitation blue LEDs with one Hamamatsu S5821-03 photodiode per optical channel. The instrument configuration and data are stored in the unit and can be transferred at any time to and from a laptop computer via the RS232 or USB ports, using our Windows-based software.

The measured phase shift (φ) and the luminescence lifetime (τ), for a particular modulation frequency of the excitation light source (*f*), are related through the well-known Equation (2) [40]:

(2) tanϕ=2πfτ

If the emission decay is perfectly exponential (e.g., in isotropic solutions of the luminophore), the observed φ can be used to determine the τ instead of the single photon timing technique. However, if the excited state decay is an ensemble of luminophore molecules in different microenvironments, the value of τ calculated from φ is an averaged one, which also depends on the modulation frequency and is not always coincident with the pre-exponentially weighted emission lifetime determined from SPT (see Section 3.4) [41]. All spectroscopic, emission lifetime, and luminescence phase shift measurements have been performed on fully cured (>5 days) polymer materials (see below) unless otherwise stated.

Temperature sensing with the phase shift-based luminometer was carried out in two ways: on the one hand, the temperature-sensitive polymer layers (see below) were attached to the optical fiber tips, for realizing environmental/industrial monitoring instrumentation. On the other hand, sensitive layers were deposited into organ-on-a-chip devices for monitoring the internal temperature from the outside with the fiberoptic luminometer.

### 2.6. Temperature Control

For the macroscopic monolithic sensor tips, temperature was kept within ±0.25 °C with a digital water bath/circulator (mod. 9110, Polyscience, Niles, IL, USA). When working at temperatures below 0 °C, a water/ethylene glycol (1:1 *v*/*v*) mixture was employed as cryogenic fluid. For testing the microscopic temperature-sensitive films, temperature was controlled with a heating plate (IKA^®^ RCT basic, Staufen, Germany) and monitored with a commercially available thermistor (LM335, Texas Instruments, Dallas, TX, USA).

### 2.7. Fabrication of the Sensor Tips

A copolymer of EHMA and MMA doped with the temperature indicator dye was prepared by dissolving 0.23 mg of [Ru(phen)_2_(4-Clp)]Cl_2_ in 0.1 mL of EHMA, and mixing the solution with 3 mL of MMA containing 0.23 mg of AIBN. Round-bottomed glass tubes (50 × 6 mm^2^, 5 mm i.d.) were filled with ca. 0.5 mL of the mixture, sonicated for 10 min and heated at 60 °C for 72 h. A piece of ca. 3 mm from the bottom of the Ru-doped polymer matrix was cut after drawing the cured material out of the mold, polishing several times with sand paper of increasingly fine grain the flat side to be in contact with the optical fiber.

Sol-gel glasses were produced from a mixture of ethanol (1.2 mL), deionized water (0.56 mL), and tetraethoxysilane (1 mL), after increasing the drying temperature up to 700 °C with a laboratory oven (0.5 °C/min). After formation of the monolith, differential absorbance measurements of the supernatant showed that 4 mol of the ruthenium complex, dissolved in ethanol, was incorporated into 300 mg of this porous matrix (72 h contact time).

Sensor tips prepared from a commercial epoxy resin (see above) were fabricated by dissolving ca. 0.2 mg of the dye in 1.5 mL of 2-butanone (99%, Merck, Darmstadt, Germany, DE), and adding the same amount of the hardener and the epoxy components to this solution. Borosilicate round-bottomed glass tubes were filled with this mixture, which was allowed to polymerize at room temperature for 72 h. The Ru-doped monoliths were cut and polished, as described before for EHMA-MMA copolymer.

Poly(ethyl cyanoacrylate) (PCA) monoliths containing the temperature indicator complex were prepared by filling the above mentioned borosilicate molds with ca. 500 μL solution of the Ru(II) complex in the monomer (0.20–0.35 mg in 4 mL). Due to the relatively large solution volume, CA polymerizes slowly, so that changes in the luminescence from the dyed material were observed over the first five days (see Results and Discussion section). Therefore, all of the monoliths were allowed to cure for at least five days to obtain sensors showing a stable response. After this period, the borosilicate tube was broken to extract the solid matrix. Then it was cut to ca. 1.5 mm and thoroughly polished according to the procedure described above to yield 1-mm high domed monoliths. The latter are placed in direct contact with the distal end of the optical fiber, mounted in home-made stainless-steel ferrules after covering the outermost part of the monolith with aluminum foil to eliminate the interference of the external light on the luminescence from the dyed polymer (Figure S1, Supplementary Materials). The application of a black painting overcoat was also successfully tested.

### 2.8. Temperature-Sensing Film in Microfluidic Devices

Organ-on-a-Chip devices were kindly provided by Micronit Microtechnologies (Enschede, The Netherlands) (https://www.micronit.com/products/organ-on-a-chip.html). These chips consist of three layers: a thin glass membrane-carrier sandwiched between two glass slides equipped with integrated gaskets (Figure S2a, Supplementary Materials). The gaskets are made of a perfluorinated elastomer, and are fabricated by means of a dispensing computer numerical control machine. The flow chambers are created by mechanically compressing the three layers using the dedicated (but customizable) Micronit Fluidic Connect Pro clamp (Figure S2c, Supplementary Materials). This sealing strategy yields flow channels without the need of thermal or chemical processes, and allows for re-opening the chips at any time just by opening the clamp. Temperature-sensitive [Ru(phen)_2_(4-Clp)]^2+^-dyed PCA films, with a thickness lower than 100 μm to match the size of the channel, are deposited in the internal part of the top glass slide, as described below. Sheets of polyimide tape (Kapton^®^, DuPont) of 50 µm thickness were cut into 15 mm × 45 mm pieces with three holes (4 mm dia.) along the long axis, using a cutting plotter (Graphtec CE6000-60, Irvine, CA, USA). The patterned tape sections were then transferred onto the glass slide and used as a deposition mask. Solutions of the dye (67 μM) in the pure monomer were knife-coated onto the Kapton^®^ tape-coated glass, and the tape was immediately removed leaving behind three round-shaped spots of dyed solution. In some cases, the latter were covered with a piece of 15 µm-thick aluminum foil, which remained glued onto the dyed cyanoacrylate film. The luminescent spots were allowed to cure for 24 h and fiber-optic interrogation was always made from the uncovered side of the polymer film (Figure S2b, Supplementary Materials).

## 3. Results and Discussion

### 3.1. Indicator Dye Selection

The development of a novel indicator-based luminescent sensor involves an initial stage of selecting the optimum dye for a suitable analyte detection. To that aim, six Ru(II) complexes containing different chelating ligands, namely [Ru(bpy)_3_](PF_6_)_2_, [Ru(bpz)_3_](PF_6_)_2_, [Ru(phen)_3_](PF_6_)_2_, [Ru(dpp)_3_]Cl_2_, [Ru(phen)_2_(4-OHp)](PF_6_)_2_, and [Ru(phen)_2_(4-Clp)]Cl_2_ (Scheme II), were chosen. The electronic features of the selected coordination ligands span from the significant σ-donor ability of 4-OHp to the highly π-accepting bpz. The intermediate σ-donating properties of the other chelating ligands decrease in the sequence bpy > phen > dip > 4-Clp, while their π-accepting character increases from bpy to 4-Clp. The luminescence decay profile of these complexes was recorded as a function of temperature to establish the influence of the structure and number of specific polypyridyl ligands on the ^3^MLCT excited state deactivation kinetics of the corresponding metal complexes. The emission decays in propylene carbonate (PC) could be fitted to single-exponential functions, except that shown by the [Ru(bpz)_3_]^2+^ complex for which a biexponential fit had to be used at temperatures above 35 °C. Moreover, an exponential fit could not be recovered after cooling down the solution containing this dye, pointing out to an irreversible thermal transformation of the luminescent indicator in the organic solvent. The known instability of [Ru(bpz)_3_]^2+^ is attributable to the very weak σ-donation properties of the bipyrazine when compared to either bipyridine or phenanthroline ligands [42,43]. The weak ligand field strength of bpz and related ligands approaches the ^3^MLCT and the (dissociative) ^3^MC excited states, with deleterious results for the luminescent probe design.

**Figure 3:**
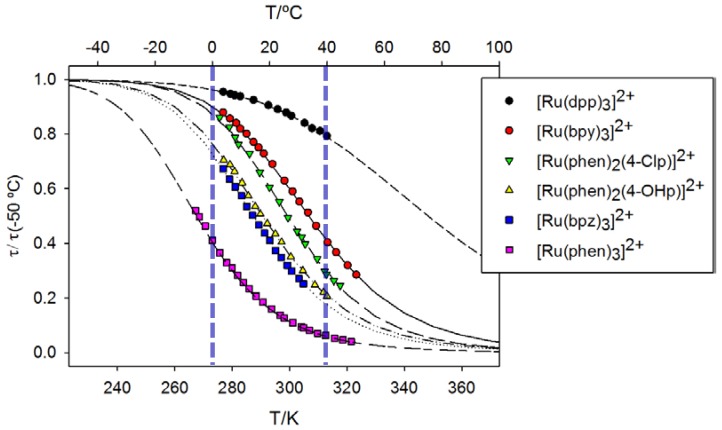
Luminescence lifetimes (normalized at 223 K) as a function of temperature of the Ru(II) complexes in deoxygenated propylene carbonate solution (PC-11 N aq HCl 10:1 *v*/*v* for the hydroxylated complex). The black lines stand for the best computer fit (Sigma Plot 11.0) of the experimental data to Equation (1), assuming contribution of only one thermally activated pathway. From top to bottom: (–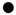
–) [Ru(dpp)_3_]^2+;^ (–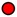
–) [Ru(bpy)_3_]^2+^; (–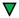
–) [Ru(phen)_2_(4-Clp)]^2+^; (–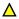
–) [Ru(phen)_2_(4-OHp)]^2+^; (–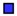
–) [Ru(bpz)_3_]^2+^; (–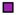
–) [Ru(phen)_3_]^2+^. The blue vertical dashed lines highlight the range 0–40 °C range of interest for the sought applications.

Fitting the experimental emission lifetimes obtained at different temperatures (Figure 3) to Equation (1) allows determination of the parameters of the fit (Table 1).

Due to the significant covariance observed between the *B* and *ΔE* parameters of the fit, they cannot be analyzed separately: only their order of magnitude and the rate constant of the temperature-activated crossing (*k*_t_ = *B*exp(‒*ΔE*/*kT*)) are meaningful quantities [44]. In this way, *B* > 10^12^ s^−1^ and *ΔE* > 3000 cm^−1^ values (at 298 K) have been attributed to thermal population and decay via the ^3^MC excited state, whereas *B* < 10^8^ s^−1^ and *ΔE* < 800 cm^−1^ indicate a non-radiative deactivation via the higher-lying (fourth) ^3^MLCT state [45]. Taking these values into account, the collected data for the Ru(II) luminophores investigated in this work (Table 1) indicate the predominance of the former deactivation pathway in all cases. The long emission lifetime of the [Ru(dpp)_3_]^2+^ dye, together with the low efficiency of its thermally-activated deactivation process (η*_t_*), probably mean that the observed decrease of its luminescence upon increasing temperature is due to both decay pathways. This fact would explain the intermediate values of *B* and *ΔE* obtained for this dye. The conjugated aromatic structure of dpp increases its π-acceptor features when compared to the unsubstituted phen ligand, leading to stabilization of the metal complex ^3^MLCT excited state with respect to its ligand-field ^3^MC level and, therefore, displays longer emission lifetimes and much lower temperature sensitivity. Nevertheless, [Ru(bpy)_3_]^2+^ and [Ru(phen)_3_]^2+^ are the ruthenium complexes most used in the literature for temperature optosensing, probably taking advantage of the vast amount of information about their luminescent features put forward for fiber-optic O_2_ measurements with these dyes [15]. Interestingly, [Ru(phen)_3_]^2+^ is the most sensitive amongst the investigated dyes in the −30 to +20 °C temperature range. However, above 20 °C its luminescence is appreciably quenched so that, at 25 °C, the efficiency of thermally activated promotion from the ^3^MLCT excited state is the lowest amongst the values obtained in this study.

The photoexcited [Ru(bpz)_3_]^2+^ is the most sensitive complex in the temperature range of interest due to its high η_t_ value. Unfortunately, this efficient promotion to the dark ^3^MC state leads to fast photodecomposition, making it unsuitable for luminescent temperature sensing. The luminescence lifetime of [Ru(phen)_2_(4-OHp)]^2+^ also shows a strong dependence with temperature but only in acidic PC (Table 1) due to the almost complete loss of its luminescence when deprotonated. The high η_t_ value found for this indicator (Table 1), close to that of [Ru(bpz)_3_]^2+^, also leads to a high photolability. Similarly to the pyrazine moieties (see above), but in a lower degree, the electron withdrawing effect of the chlorine atom on the phenanthroline moiety leads to stabilization of the ^3^MLCT excited state in [Ru(phen)_2_(4-Clp)]Cl_2_. However, this complex also contains two unsubstituted phen ligands coordinated to the metal core, the strong σ-donation of which results in destabilization of its ^3^MC level (strong ligand field). Therefore, this metal complex shows good photostability as a consequence of its high *ΔE* value (4215 cm^−1^). At the same time, its very large pre-exponential factor (1.5 × 10^14^ s^−1^) leads to significant temperature dependence, making it the best choice for a lifetime-based luminescent temperature indicator dye to be used both in the 0 to 50 °C range (water monitoring applications) [46], and the 25 to 40 °C range (cell culture applications) [47,48].

### 3.2. Photophysical Characterization of [Ru(Phen)_2_(4-Clp)]^2+^ in Solution

The absorption and emission maxima of [Ru(phen)_2_(4-Clp)]^2+^ in different organic solvents and in water, together with its luminescence lifetimes in air-equilibrated and deoxygenated solutions, are summarized in Table 2. The selected organic solvents try to simulate the environment around the indicator dye in the best polymer matrix (see Section 3.3). The absorption spectra show intense UV bands corresponding to intraligand (IL) π → π* transitions and a broad band, with two maxima in the visible region, assigned to a metal-to-ligand charge transfer transition (d → π*) [31]. No significant solvent effect has been observed either on the maxima or on the intensity of the absorption bands within the relatively polar solvents used in this work. This complex also displays, as expected, an unstructured emission band centered at 610–619 nm, depending on the solvent polarity and viscosity. The differences found in the emission lifetime as a function of the solvent can be explained in terms of the corresponding oxygen solubility (higher in organic solvents), solvent viscosity, and energy transfer to the solvent O–H oscillators.

### 3.3. Polymer Sensor Tip Development

After photophysical characterization of the selected Ru(II) dye in solution, four polymeric matrices were tested in order to find the best solid support for the dye immobilization without altering its temperature-sensing features. A facile manufacturing of the dye-doped polymer material, together with a good thermal conductivity and low oxygen permeability were the main searched characteristics for the temperature sensor matrix. The investigated polymers were a sol-gel material, an epoxy resin, poly(ethyl cyanoacrylate) (PCA), and a copolymer of an hydroxylated acrylate monomer (EHMA) and MMA (see Experimental section). The scarce solubility of the Ru(II) complex in pure MMA led us to copolymerize this monomer with its hydroxylated derivative where the dye was soluble in.

The temperature sensitivity of the dye entrapped in the polymeric supports, in terms of the relative change in the luminescence intensity displayed by the manufactured sensor tip when temperature was dropped from 40 to 0 °C, has been summarized in Table 3. The intensity change for other temperatures is shown in Figure S3 (Supplementary Materials).

**Figure 4:**
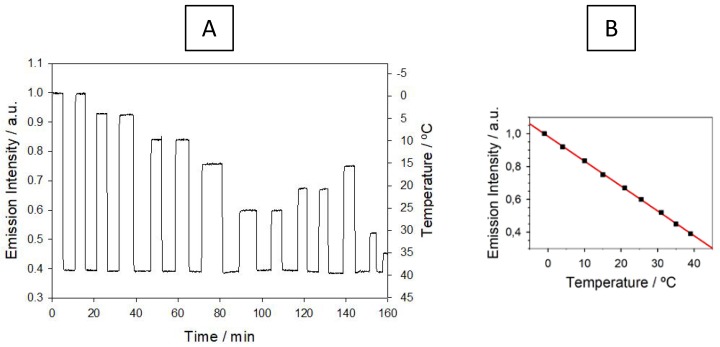
Response of the luminescent temperature sensor containing [Ru(phen)_2_(4-Clp)]^2+^ immobilized into poly(ethyl cyanoacrylate). (**A**) Real-time response; (**B**) Calibration plot.

Supports containing hydroxyl groups in their structure, such as the sol-gel and p(EHMA-MMA), showed a lower temperature sensitivity, probably due to competitive deactivation of the luminophore via energy transfer to the surrounding O–H oscillators. Moreover, the sensor tip manufactured with the epoxy resin displayed a slower and unstable response to temperature changes, owing to a lower thermal conductivity. On the contrary, luminescently-doped polycyanoacrylate monoliths showed a fast and stable response (Figure 4), allowing, at the same time, fabrication of readily-made reproducible sensor tips.

### 3.4. Photophysical Characterization of the Dye Entrapped into a Poly(Cyanoacrylate) Matrix

Different authors have studied the effect of the polymer support on the photophysical properties of Ru(II) complexes entrapped into rigid matrices. Thus, it has been demonstrated the inhibition of the emission decay through the ^3^MC pathway for photoexcited Ru(bpy)_3_^2+^ (and related complexes) immobilized in cellulose acetate [43], zeolites [49], or metal-organic frameworks (MOFs) [50,51], so that its small temperature dependence in the region of interest is then controlled by the additional (fourth) MLCT state. This effect is due to the anti-bonding nature of the excited state ^3^MC, because its population is expected to lead to the elongation of the Ru-N bonds that is inhibited in the rigid medium.

**Figure 5:**
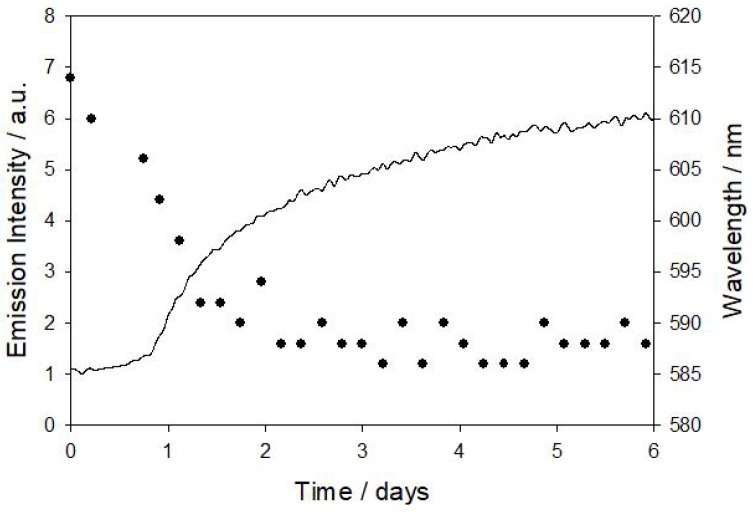
Continuous monitoring of the luminescence intensity (solid line) and maximum emission wavelength (full circles) of the [Ru(phen)_2_(4-Clp)]^2+^ complex dissolved in ethyl cyanoacrylate during polymerization at room temperature.

We have monitored the changes of the luminescence spectrum of [Ru(phen)_2_(4-Clp)]^2+^ dissolved in the cyanoacrylate monomer during its polymerization. The metal complex displayed a blue shift of its emission band as the monomer became harder (during the first three days, Figure 5). The emitting ^3^MLCT cannot be stabilized by solvent reorganization in the solid matrix (rigidochromism), and, therefore, the radiative deactivation takes place from a more energetic excited state leading to the emission blue shift. The luminophore also undergoes a strong enhancement of its luminescence intensity (Figure 5), largely due to the less accessibility of the oxygen to the indicator, which decreases the deactivation pathway that is caused by this effective quencher. The observed changes in the intensity and maximum emission wavelength of the dye dissolved in the monomer also allow for a continuous monitoring of the polymerization process to assess its progress.

Table 4 lists the emission lifetimes obtained from the analysis of the luminescence decay kinetics of the [Ru(phen)_2_(4-Clp)]/PCA sensor tip. Under the two evaluated temperatures (0.0 and 40.0 °C), the emission decays could only be fitted correctly to 3-exponential functions. Several environments for the dye into the solid matrix are the origin of the different lifetimes found: the longest one is associated to very diluted ruthenium complexes entrapped in the rigid polymer medium, while the shortest one corresponds to aggregated dye molecules dwelling in other regions of the polymer monolith. The bright aggregates undergo nonetheless self-quenching of their emission lifetime, as it has been shown by luminescence lifetime imaging microscopy in O_2_-sensitive polymer layers that are also manufactured with Ru(II) complexes [52]. Actually, the situation is probably more complex and the number of luminophores in different microenvironments of the polymer matrix is expected to be higher than just three. Once the 3-exponential function provides an excellent fit, we do not try a larger number of exponential terms. Intermediate lifetimes correspond to populations of Ru(II) dye molecules separated by various distances or surrounded by different domains generated by the PCA chains.

### 3.5. Temperature Measurements Based on the Luminescence Lifetime of the Polymer Material

**Figure 6:**
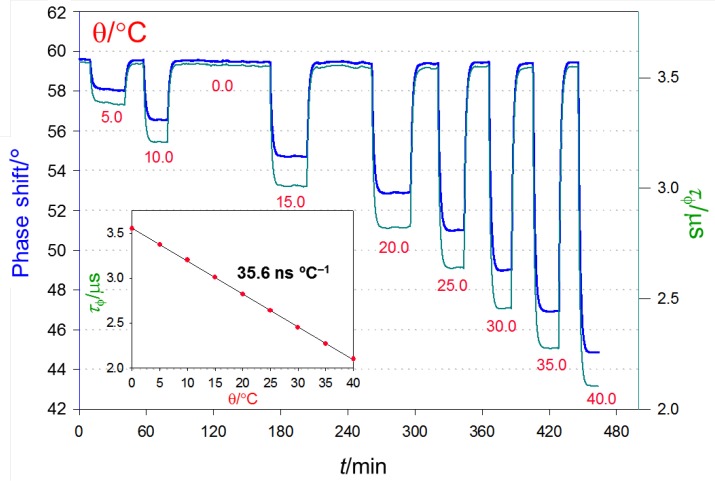
Temperature response of the Ru(phen)_2_(4-Clp)^2+^-doped poly(ethyl cyanoacrylate) luminescent optode fabricated with an initial concentration of dye in ethyl cyanoacrylate of 29 μM. The thick blue line represents the experimental raw data (phase shift) measured with the fiber-optic luminometer (excitation LED modulation *f* = 78 KHz). The corresponding emission lifetime (thin green line and inset), i.e., the experimental (average) value obtained from the phase shift angle ($\tau_{\phi }=\tan\phi /2\pi f$) displays a linear response in the 0 to 40 °C range (*r* = 0.99987), with a slope of 35.6 ns °C^−1^.

Macroscopic Temperature-Sensitive Optical Fiber Sensor. After evaluating the temperature optode response by steady-state emission measurements, the optical sensor was evaluated with a fiber-optic phase-sensitive luminometer. To that end, the sensor tips were coated with aluminum foil for protection against the income of external light to the detector, and placed at the end of a single optical fiber (Figure S1). The sensitive terminal was introduced in a temperature-controlled water bath and the emission phase shift, as generated by the luminophore lifetime was continuously recorded (Figure 6). This emission lifetime decreases linearly with temperature in the tested range (*r* = 0.99987), simplifying the optode calibration. A continuous monitoring of the emission intensity was simultaneously carried out thanks to the value of the amplitude of the modulated luminescence waveform provided by the instrument. Intensity values confirm the dye photostability during the measurements.

The optimal modulation frequency of the blue light source (78 kHz) was chosen to provide the maximum difference (14.3°) between the phase angles that were measured at 0 and 40 °C. This temperature range is the most useful for water courses monitoring, the sought application for our luminescent sensor. The apparent small discrepancy between the emission lifetimes of Ru(phen)_2_(4-Clp)^2+^ embedded in PCA calculated from the observed phase shift (τ_φ_, Figure 6) and those calculated from the multiexponential decay of the luminescence (τ_M_, Table 4) is simply due to the different weight of the short and long components (τ_i_) in the τ_M_ calculation and in the overall phase shift. While the former depends on $\alpha_{i}\tau_{i}$, the latter rather depends on the modulation frequency, according to $\alpha_{i}\tau_{i}/\left[1+{\left(2\pi f\right)}^{2}\tau_{i}^{2}\right]$ [41] (pp. 191–192). Therefore, higher modulation frequencies yield shorter τ_φ_ values due to the heavier contribution of the shorter-lived components.

The effect of the dye loading in the polymer was also investigated. We found a modest 7% increase of φ (0 °C) in going from an initial concentration of Ru(phen)_2_(4-Clp)^2+^ in ethyl cyanoacrylate of 29 to 67 μM, accompanied by a 15% decrease of φ (40 °C); higher levels of doping (up to 114 μM) lead to negligible further increase of φ (0 °C), while significantly increasing φ (40 °C). Those variations translate into phase shift excursions in the 0–40 °C range of 14.3° at 29 μM, to 22.6° at 67 μM. More concentrated initial solutions actually yield smaller excursions of φ probably due to larger populations of aggregated dye species and the self-quenching of the photoexcited dye lifetime they undergo (see Section 3.4).

The response time of the temperature luminescent sensor is determined by the calibrated thermostatic circulator change rate. Nevertheless, instantaneous transfer of the fiber-optic sensor tip from an ice-water bath at 0 °C to the thermostatic bath at 40 °C led to response times on the order of 20 s (*t*_90%_). The optode response can be accelerated using thinner PCA/dye monoliths attached to the optical fiber at the expense of a slightly lower sensitivity in the investigated temperature range.

The optode resolution (*R*), calculated from the calibration slope (*s*) and the standard deviation, *σ*_n−1_, for the worst signal-to-noise ratio (measured at 40.0 °C), was found to be 0.05 °C under the optimized conditions (*R* = 3σ_n__−1_/*s*, n = 20). The sensor tip reproducibility and its response repeatability were better than 2% and 1%, respectively, also evaluated from the relative standard deviation of the temperature measurements.

The effect of dissolved molecular oxygen, a well-known efficient quencher of Ru(II) luminophores (see above), on the response of the optode was also studied (Figure S4, Supplementary Materials). No significant changes were observed when dipping the sensor tip into air equilibrated or fully deoxygenated solutions at identical temperature due to the low oxygen permeability of the selected polymer (PCA). Obviously, any influence of O_2_ is suppressed for the aluminum foil-covered polymer monoliths.

**Figure 7:**
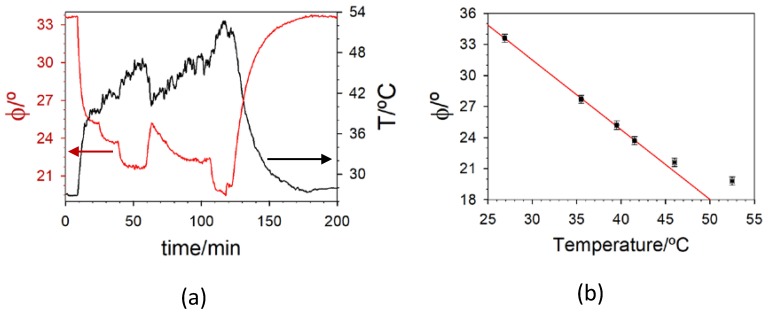
(**a**) Example of the fiberoptic luminescence-based temperature sensor response (red line). The temperature was varied with a heating plate (IKA RCT basic) and monitored with a commercially available thermistor (LM335, Texas Instrument, black line); (**b**) Phase-shift response with temperature. The red line is the linear fit of the experimental data between 26.9 and 41.5 °C (parameters of the best fit φ/° = *A* + *b T*/°C: *A* = (51.7 ± 0.3)°; *b* = (−0.674 ± 0.009)°(°C)^−1^; *r*² = 0.9996).

Microscopic Temperature-Sensitive Films inside Organ-on-a-Chip Devices. Finally, the optimized sensor was tested for temperature monitoring into organ-on-a-chip devices. The dyed polymer was deposited as thin spots and the chips were subjected to temperature variations from ca. 27 to 52 °C (Figure 7).

The response is linear between 27 and 43 °C, a suitable range for monitoring cell cultures [47]. The micrometric thickness of the sensitive spots has positive and negative consequences. On the one hand, the response time (the delay between the thermistor and the optode signals) of the temperature-sensitive film is shorter than 5 s. On the other, the thin sensor is no longer insensitive to O_2_ (see Figure S5, Supplementary Materials). However, this interference is easily suppressed by measuring that species and temperature at the same time, something that can be readily performed in a multichannel instrument, like ours. Moreover, the interference of O_2_ can be eliminated by covering the dyed PCA spot with commercial aluminum foil which, besides decreasing external light interference blocks permeation of gases without lowering the sensor sensitivity to temperature.

## 4. Conclusions

Molecular engineering of Ru(II) polypyridyl complexes allows for the design and manufacture of luminescent temperature sensors that outperform those described up to date based on standard Ru(II) indicator dyes for oxygen monitoring. In this way, a novel heteroleptic complex with two 1,10-phenanthroline and one 4-chloro-1,10-phenanthroline ligands embedded in poly(cyanoacrylate) provides optimum temperature response and photostability (at least) in the 0–50 °C range, using both emission intensity- or lifetime-based measurements with commercially-available instrumentation for optical O_2_ sensing. Moreover, this polymeric material provides affordable, easily made, and reproducible sensitive elements with excellent optical quality that can be tailored to the sought application.

The applications of polymer-based luminescent temperature sensors doped with Ru(II) complexes are countless and not only restricted to those illustrated here, namely temperature monitoring of surface waters in combination with other Ru(II)-polypyridyl-based optodes (O_2_, pH, CO_2_, NH_3_, Cu^II^, etc.) [10] plus a multichannel field-deployable optoelectronic unit, and cell culture monitoring with lab-on-a-chip systems. Other uses where such polymeric optical sensors may provide a definitive advantage are currently being investigated, e.g., temperature monitoring in bioreactors, aerospace applications, and temperature measurements within cells and tissues (the latter upon encapsulation of the indicator dye in polymer nanocapsules).

## Figures and Tables

**Table 1:** Kinetic parameters for the ^3^MLCT excited state deactivation of the investigated Ru(II) complexes in deoxygenated propylene carbonate, obtained from fitting the experimental emission lifetime data as a function of temperature to Equation (1) (see text).

Complex	*A* × 10^3^ /s^−1^	*B* × 10^11^ /s^−1^	Δ*E* /cm^−1^	*r*	τ/μs ^a^	*k_t_* × 10^−5^ /s^−1 a,b^	η*_t_* ^a,c^
[Ru(phen)_3_]^2+^	235 ± 5	446 ± 89	3546 ± 23	0.9998	0.53 ± 0.01	1.6	0.08
[Ru(dpp)_3_]^2+^	146 ± 1	0.2 ± 0.1	2861 ± 162	0.9993	6.0 ± 0.1	0.2	0.14
[Ru(bpy)_3_]^2+^	656 ± 8	481 ± 219	3858 ± 100	0.9996	0.96 ± 0.02	3.9	0.37
[Ru(phen)_2_(4-Clp)]^2+^	236 ± 4	1500 ± 937	4215 ± 134	0.9995	2.22 ± 0.04	2.2	0.48
[Ru(phen)_2_(4-OHp)]^2+ d^	276 ± 10	347 ± 228	3764 ± 141	0.9995	1.40 ± 0.03	4.4	0.62
[Ru(bpz)_3_]^2+^	388 ± 10	753 ± 344	3808 ± 96	0.9998	0.87 ± 0.02	7.8	0.67

^a^ At 298 K; ^b^ *k*_t_ = *B* exp(−*ΔE*/*kT*); ^c^ Efficiency of the thermally-activated promotion from the ^3^MLCT excited state to the ^3^MC: η*_t_* = *k*_t_/(*k*_r_+ *k*_nr_+ *k*_t_) = *k*_t_τ (see Figure 1); ^d^ In propylene carbonate–aq. HCl (11 N), 10:1 *v*/*v*.

**Table 2:** Spectroscopic features and emission lifetimes of [Ru(phen)_2_(4-Clp)]^2+^ in solution at 25.0 °C.

Solvent	Δλ_max_^abs^/nm (ε/M^−1^ cm^−1^) ^a^	Δλ_max_^em^ /nm ^b^	τ_Air_ /μs ^c^	τ_Ar_ /μs ^c^
water	224 (59,500), 262 (83,000), 425 (13,900), 445 (14,100)	610	0.82	1.49 ^d^
propylene carbonate	224, 262, 425, 445	619	0.51	2.22
acetonitrile	224, 262, 425, 445	612	0.24	1.90
butyronitrile	224, 262, 425, 445	610	0.34	1.63

^a^ Uncertainty ± 1 nm; the uncertainty for ε is ± 10%; ^b^ Uncorrected for the instrumental response; uncertainty ± 2 nm; ^c^ Uncertainty 2%; ^d^ Luminescence quantum yield (Φ_em_) under these conditions: (0.12 ± 0.01).

**Table 3:** Temperature sensitivity of the luminescence of [Ru(phen)_2_(4-Clp)]^2+^ immobilized into different polymers.

Polymer support ^a^	PCA	EPOXY	SOL-GEL	p(EHMA-MMA)
*I*_0_/*I*_40_^b^	3.6	3.3	2.2	1.8

^a^ PCA: poly(ethyl cyanoacrylate). See Experimental section for details on the sensor materials; ^b^ Relative emission intensity at 0.0 and 40.0 °C (λ_em_ = 590 nm).

**Table 4:** Luminescence lifetimes of [Ru(phen)_2_(4-Clp)]^2+^ in solution and immobilized into the poly(ethyl cyanoacrylate) matrix. The emission decays of the indicator dye have been fitted to the function $I\left(t\right)=A_{1}e^{-t_{1}/\tau }+A_{2}e^{-t_{2}/\tau }+A_{3}e^{-t_{3}/\tau }$.

*T*/°C	Δτ_PC_/μs ^a^	τ_1_/μs (α_1_) ^b^	Δτ_2_/μs (α_2_) ^b^	Δτ_3_/μs (α_3_) ^b^	*τ_M_* ^c^	Δτ_φ_^d^
0.0	3.71	5.78 (0.44)	3.03 (0.36)	1.27 (0.20)	3.89	3.51
40.0	1.23	3.35 (0.30)	1.19 (0.45)	0.18 (0.25)	1.59	2.08

^a^ Lifetime in deoxygenated propylene carbonate (PC) solution; ^b^ The α values represent the relative pre-exponential term ($\alpha_{i}=A_{i}/\sum_{i}A_{i}$); ^c^ Pre-exponentially weighted emission lifetime $\tau_{M}=\sum_{i}\alpha_{i}\tau_{i}$; ^d^ Average emission lifetime determined from the experimental phase shift (φ at *f* = 78 KHz): $\tau_{\phi }=\tan\phi /2\pi f$ [41].

## Supplementary Materials

The following items are available online at http://www.mdpi.com/2073-4360/10/3/234/s1, Figure S1: Picture of the temperature-sensitive optical fiber tips, Figure S2: Drawings/pictures of the 3-layer chip, temperature-sensitive spots into Micronit’s organ-on-a-chip device, and of the Fluidic Connect PRO chip holder with fluidic connections and optical fibers, Figure S3: Variation with temperature of the luminescence intensity from the dye-doped polymer monoliths, Figure S4: Response to O_2_ of the [Ru(phen)_2_(4-Clp)]/PCA temperature-sensitive optical fiber tip, Figure S5: Response to O_2_ of the [Ru(phen)_2_(4-Clp)]/PCA temperature-sensitive spots in the organ-on-a-chip device.
