# Supplementary material for: Optimization of Temperature Sensing with Polymer-Embedded Luminescent Ru(II) Complexes
Source: Polymers (Basel). 2018 Feb 26;10(3):234. doi: 10.3390/polym10030234 (PMC6414956; doi:10.3390/polym10030234)
Supplement: Supplementary file 1 [file polymers-10-00234-s001.pdf]

Supplementary Material

Optimization of Temperature Sensing with Polymer-Embedded Luminescent Ru(II) Complexes

Nelia Bustamante[#], Guido Ielasi, Maximino Bedoya, and Guillermo Orellana*

Chemical Optosensors and Applied Photochemistry Group (GSOLFA), Department of Organic Chemistry, Faculty of Chemistry, Universidad Complutense de Madrid, E-28040 Madrid, Spain; nbustamante@aenor.com (N.B.); gielasi@ucm.es (G.I.); mabedoya@ucm.es (M.B.).

[#] Current address: AENOR, Génova, 6, 28004 Madrid (Spain)

***** Correspondence: orellana@quim.ucm.es; Tel.: +34-913944220

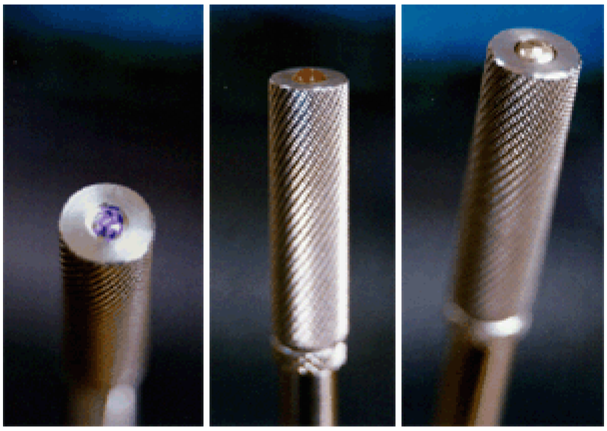

Figure S1. Optical fiber tip with the $[Ru(phen)_2(4\text{-}Clp)]^{2+}$/PCA sensitive material. Left: top view showing the blue light excitation; center: side view showing the red luminescence from the polymer terminal; right: polymer terminal covered with aluminum foil.

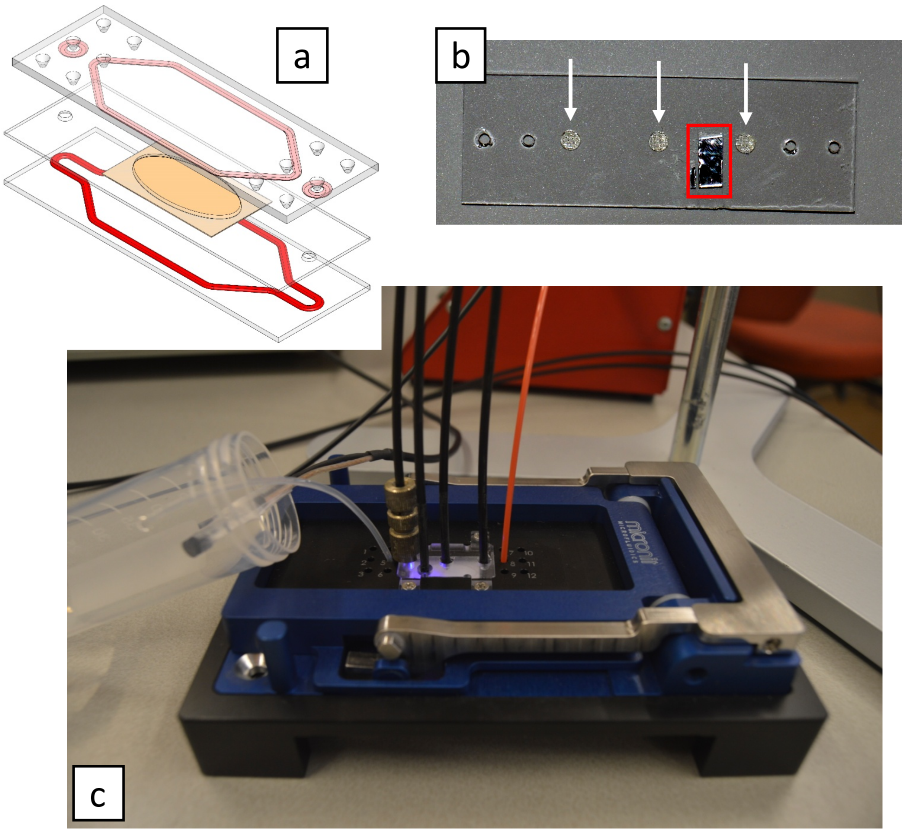

Figure S2. a) Micronit 3-slide chip scheme, with the thin glass supporting the permeable membrane (in orange) sandwiched between two glass slides carrying the elastomeric gaskets (in red). b) Top glass slide (before the gasket deposition) with three naked T-sensitive luminescent spots (white arrows) and one aluminium foil-covered T-sensitive luminescent spot (red square). c) The Micronit Fluidic Connect PRO chip holder (ref. FC_PRO_CH4515) with the fluidic inlet (orange tube) and outlet (transparent tube) and the 4 optical fibers attached.

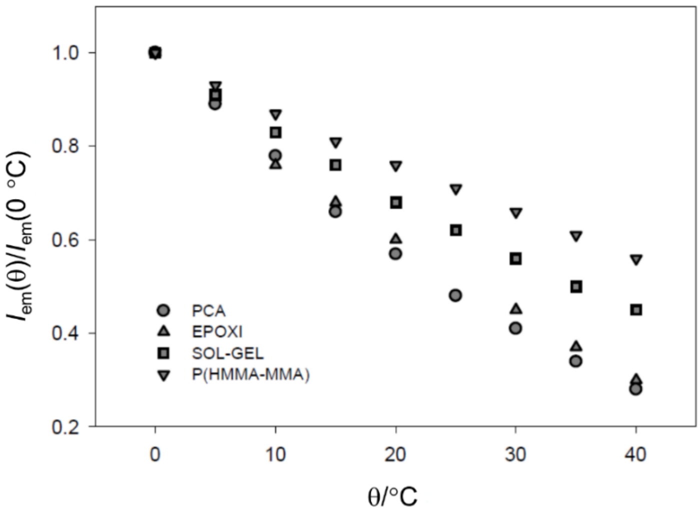

Figure S3. Variation with temperature of the luminescence intensity at 590 nm of the polymer monoliths doped with $[Ru(phen)_2(4\text{-}Clp)]^{2+}$ (see Experimental section).

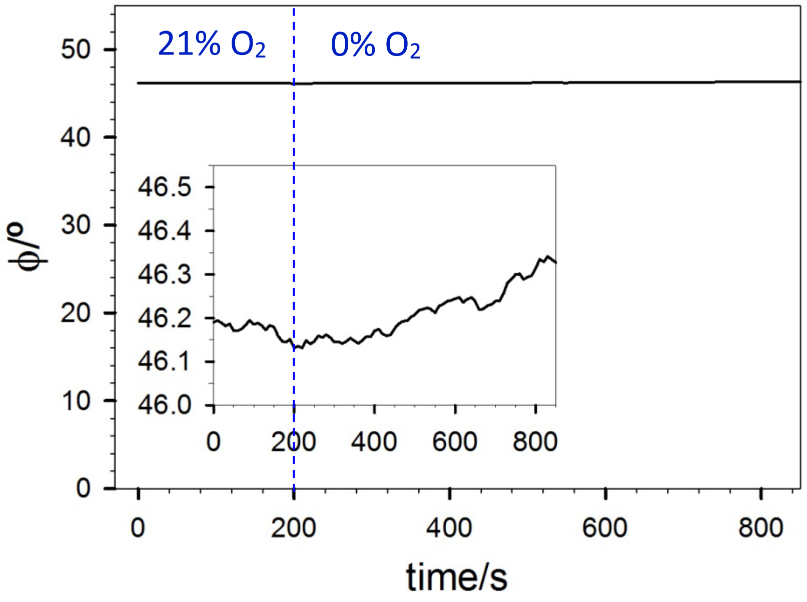

Figure S4. Response to O_2 (from 21% to 0% O_2 in N_2 by volume) of the [Ru(phen)$_2$(4-Clp)]/PCA temperature-sensitive optical fiber tip at 25 °C. The inset shows a zoom of the y-axis of the plot.

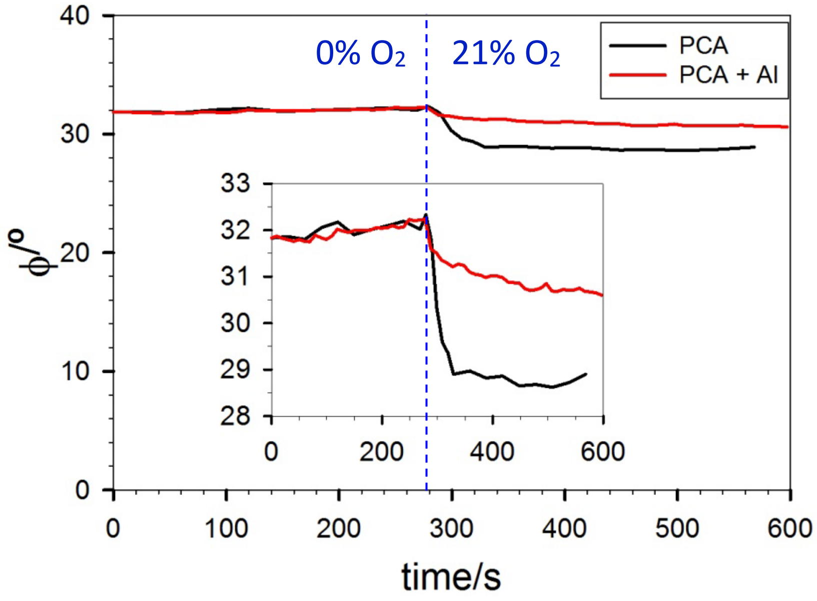

Figure S5. [Ru(phen)$_2$(4-Clp)]/PCA temperature-sensitive spots in the organ-on-a-chip device: response to O_2 (from 0% to 21% O_2 in N_2 by volume) at 27 °C. Black line: naked T-sensitive luminescent film; red line: aluminum foil-covered T-sensitive luminescent film.
